# Optimization of Contact Pad Design for Silver Nanowire-Based Transparent Heater to Improve Heating Characteristics

**DOI:** 10.3390/nano14211735

**Published:** 2024-10-29

**Authors:** Seo Bum Chu, Yoohan Ma, Jinwook Jung, Sungjin Jo, Dong Choon Hyun, Jae-Seung Roh, Jongbok Kim, Dongwook Ko

**Affiliations:** 1 Department of Materials Science and Engineering, Kumoh National Institute of Technology, Gumi 39177, Republic of Korea; csb5453@naver.com (S.B.C.); john931023@gmail.com (Y.M.); jkim3161@gmail.com (J.J.); jsroh@kumoh.ac.kr (J.-S.R.); 2 Department of Energy Engineering Convergence, Kumoh National Institute of Technology, Gumi 39177, Republic of Korea; 3 School of Architectural, Civil, Environmental and Energy Engineering, Kyungpook National University, Daegu 41566, Republic of Korea; sungjin@knu.ac.kr; 4 Department of Polymer Science and Engineering, Kyungpook National University, Daegu 41566, Republic of Korea; dong.hyun@knu.ac.kr; 5 Advanced Materials Research Center, Kumoh National Institute of Technology, Gumi 39177, Republic of Korea

**Keywords:** transparent heater, silver nanowire, contact pad, joule heating

## Abstract

Transparent heaters are gaining significant attention for applications such as antifog glass, smart windows, and smart farm greenhouses. A transparent heater basically consists of transparent conducting materials that serve as a heating area and contact pad electrode to apply power. To fabricate a transparent heater, materials with excellent light transmittance and low sheet resistance are required. Among various transparent conducting materials, such as Indium Tin Oxide (ITO), carbon nanotube (CNT), graphene, and silver nanowires (AgNWs), AgNWs are particularly favored due to their good electrical, optical, and mechanical properties. However, in order to improve the heating characteristics of transparent heaters, research is essential not only on improving the properties of transparent conducting materials but also on the design of contact pad electrodes that can uniformly improve current distribution. Here, we explore various shapes of contact pad electrodes for AgNW-based transparent heaters to improve current distribution. Shapes such as line, spot, twisted, and parallel-type contact pad electrodes are designed and investigated to optimize overall heating characteristics. We analyze the heating properties of these transparent heaters with various contact pad electrodes, demonstrating how their specific shape and size affect heating characteristics and uniformity. We also investigate the optimal shape of the contact pad electrode to minimize transmission loss through UV-VIS spectroscopy. As a result, we confirm that the shape of the contact pad electrode was important for simultaneously achieving high heating characteristics of 120 °C, good heating uniformity, and over 80% transparency in an AgNW-based transparent heater.

## 1. Introduction

Transparent heaters are made of materials combining visible light transmission with heating capabilities and are receiving widespread attention and application in various fields. Transparent heaters are used for antifog glass, smart windows, and advanced agricultural technology in smart farm greenhouses, and the utility of transparent heaters continues to expand [1,2,3,4,5,6,7,8,9]. Therefore, a transparent electrode material with constant thermal conductivity and high transmittance is used. The key characteristics of a transparent heater are high light transmittance and low sheet resistance [10,11,12]. Indium Tin Oxide (ITO) is the representative transparent electrode material that has a high light transmittance of over 90% and high conductivity. ITO has found widespread application in various optoelectronic devices, including solar cells, organic light-emitting diodes (OLEDs), displays, smart windows, and touch screens [13,14,15,16,17]. However, employing ITO is limited by properties such as low flexibility, vulnerability to price fluctuations, and nonideal thermal conductivity. Therefore, much research is being conducted to find alternative materials such as carbon nanotubes (CNTs), graphene, silver nanowires (AgNWs), conductive polymers, and composite materials with improved properties [18,19,20,21,22]. Among the new alternatives, AgNWs have emerged as a promising candidate, demonstrating high flexibility, conductivity, and significant thermal conductivity [23], as well as favorable optical properties. AgNWs stand out for their cost-effectiveness in large-area roll-to-roll processing through a solution-based process [24], offering the advantage of controllable density for adjustments in transmittance and thermal conductivity fit to specific applications.

While fabricating AgNW-based transparent heaters, it is crucial to consider not only the transparent conducting network but also the two metallic terminate side contact pad electrodes [25,26,27,28,29,30]. These pads enable good current distribution, which is essential for high heating efficiency. Additionally, uniform heating characteristics are influenced by specific current flows. Although AgNWs should be fully connected, the current distribution can remain constant due to the complexity of the AgNW networks. Therefore, many researchers working on transparent heaters opt for a linear-type contact pad to ensure good current distribution using conductive copper tape or metal contact pads deposited by thermal evaporation [31,32,33,34]. To enhance the heating temperature and uniformity of the heating characteristics, the design of the contact pad electrodes, which could enable improvement of current distribution, should be considered, along with improvements to the transparent conducting networks.

In this study, we investigated the effect of the heating performance based on the shape of the contact pad electrode. We designed the contact pad electrodes in various shapes: line, spot, twist, and parallel. We confirmed that the heating performance of the transparent heater varies with the shape of the contact pad electrode. We also explored how the shape of the contact pad electrode affects performance by controlling the width and length of the parallel-type contact pad electrode. This optimization aims to enhance the overall heating performance of the transparent heater. We confirmed uniform heating performance across the surface of the transparent heater through measurements using an infrared thermographic camera spot meter. Through UV-visible transmittance analysis, we verified that the optimized transparent heater maintains high performance even with minimal transmittance loss.

## 2. Materials and Methods

**Figure 1:**
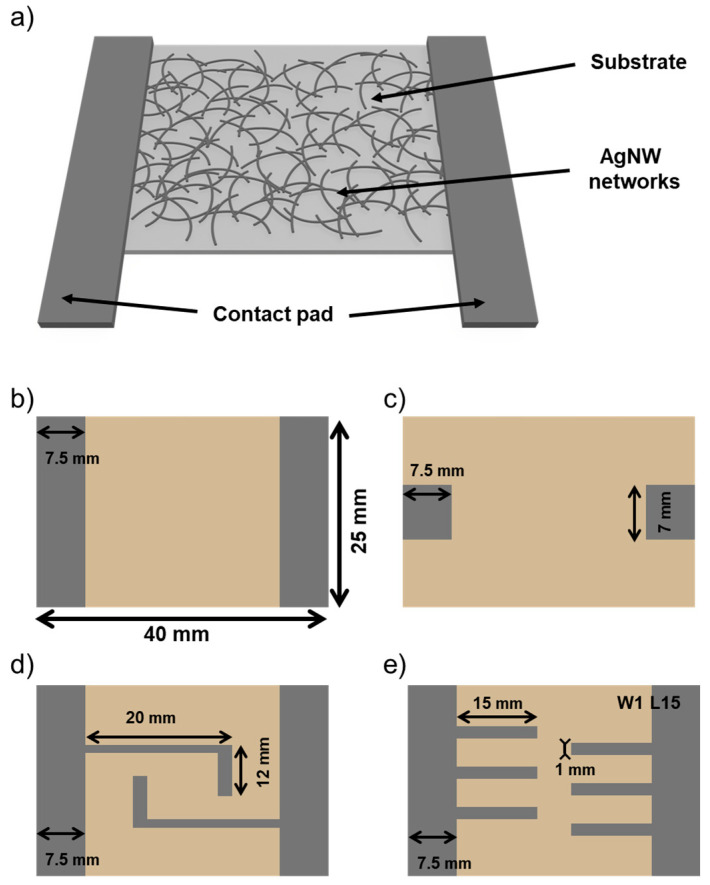
(**a**) Schematic of AgNW-based transparent heater design of contact pad electrode, (**b**) line type, (**c**) spot type, (**d**) twist type, (**e**) parallel type.

To fabricate an AgNW-based transparent heater, we use a glass substrate, AgNW networks as a heating surface, and a contact pad to efficiently apply current to the AgNW networks, as shown in Figure 1a. Before fabricating the AgNW-based transparent heaters, various shaped metal masks (linear, spot, twist, parallel-type) were prepared for deposition, as illustrated in Figure 1b–e. In addition, we investigated the characteristics of the heater by adjusting the width and length of the electrodes and observing how the shape of the finger (also referred to as a finger electrode) affects the heating characteristics of the heater. The finger widths were designed at 1, 1.5, 2, and 2.5 mm, and the finger lengths at 5, 10, 15, and 20 mm.

The transparent heater based on AgNWs was fabricated through the following process: First, 40 × 25 mm slide glass substrates were cleaned with acetone and isopropyl alcohol (IPA, Daejeong, Republic of Korea) at 60 °C for 15 min each, followed by drying at 100 °C for 10 min. Subsequently, a 0.3 wt% solution of AgNWs in deionized water (average diameter: 25 nm; length: 35 μm, C3 Nano Korea, Yongin, Republic of Korea) was dropped onto the glass substrates using 700 μL, then spin-coated at 1000 rpm for 1 min, and dried on a hot plate at 100 °C for 1 min. To deposit the specific contact pads, the designed metal mask was placed on the AgNW-coated samples, and then a 100 nm thick silver electrode was deposited through thermal evaporation.

To investigate the electrical characteristics of an AgNW-based transparent heater, the sheet resistance was examined by using a portable 4-point probe (RC2175, EDTM, Toledo, OH, USA), as shown in Figure S1. The surface morphology of the AgNW-based transparent heater was characterized using a field-emission scanning electron microscope (FE-SEM, TESCAN MAIA, Brno, Czech Republic). The density of AgNWs was calculated with image processing software (Image J 1.52d). To confirm the heating properties of the transparent heater, the prepared transparent heaters were heated for 3 min and cooled for 2 min using a source meter (Keithley 2400, Keithley, OH, USA) under conditions of 3, 5, and 7 V. The heating characteristics of the transparent heaters were analyzed using an IR-Thermo Camera (SE-A325, FLIR SYSTEMS, Portland, OR, USA), as shown in Figure S1. To check the heat uniformity across the transparent heater’s area, we measured heat at 6 spot points on the transparent heater using the IR-Thermo Camera’s spot meter function. Additionally, we analyzed the light transmittance using a UV-Vis spectrometer (UV-2600, Shimadzu, Kyoto, Japan) to investigate the influence of finger-shaped electrodes on the optical properties. The wavelength range for transmittance measurements was 300 nm to 800 nm.

## 3. Results and Discussion

### 3.1. Optimizing Electrode Shape of Transparent Heater Through Heating Characteristics

**Figure 2:**
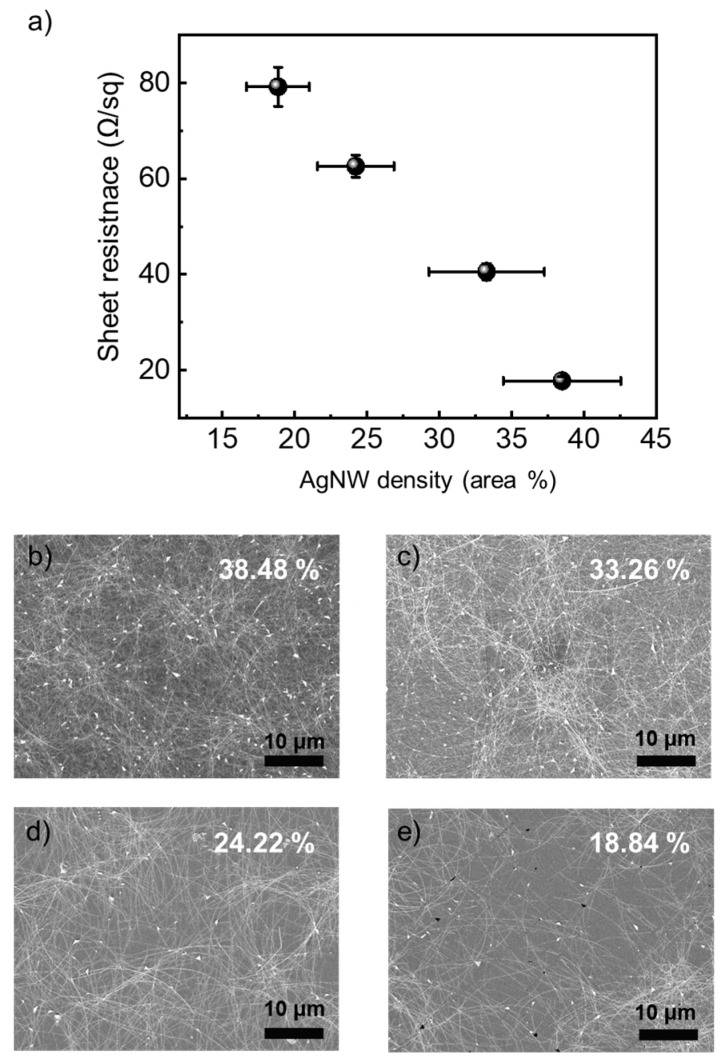
(**a**) the sheet resistance at different AgNW density and FE-SEM image at (**b**) 38.48% AgNW density, (**c**) 33.26% AgNW density, (**d**) 24.22% AgNW density, and (**e**) 18.84% AgNW density.

The heating characteristics of a transparent heater are related to the sheet resistance of the AgNWs network, as described by the Joule heating formula [35]. The heating characteristics of a transparent heater are proportional to the square of the applied voltage and inversely proportional to the sheet resistance. So, to optimize the heating characteristics of AgNW-based transparent heaters, we should control the sheet resistance of AgNW networks to improve heating characteristics. To optimize the sheet resistance of AgNWs network, we measured the sheet resistance of AgNWs networks at different AgNWs densities, as shown in Figure 2. We controlled the density of AgNWs by adjusting the coating speed. As shown in Figure 2, the AgNW-based transparent electrodes, depending on various coating speeds, were examined using FE-SEM. The density of AgNWs was also calculated using FE-SEM image processing. As coating speed decreased, the density of AgNW increased from 18.84% to 38.48%. Therefore, the sheet resistance of the AgNWs network decreased with increasing AgNWs density because electrons could more easily flow to opposite electrodes by many paths. While the sheet resistance was lowest in the case of high AgNW density, the transparency was not optimal because AgNWs could block the surface [36,37]. The AgNWs networks with sheet resistance of 40.6 Ω/sq and transmittance of 85% could be suitable as transparent heaters due to their good conductivity and transparency. Since the density of the AgNWs network was fixed, the heating characteristics vary based on the current distribution, which is influenced by the design of the contact pad. To investigate the heating characteristics of AgNW-based transparent heater with various contact pad shapes, we designed contact pad electrode shapes such as line type, spot type, twist type (T), and parallel type (P), as illustrated in Figure 1b–e.

**Figure 3:**
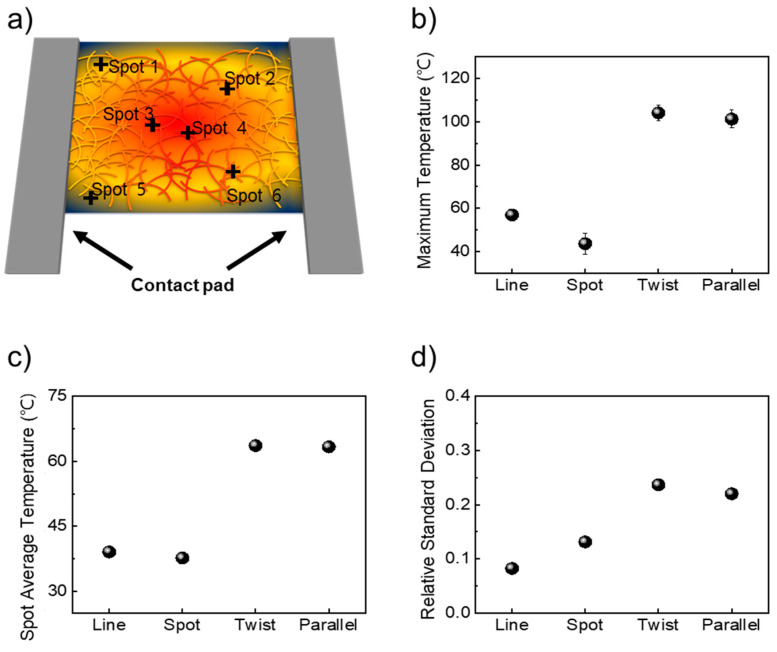
(**a**) Scheme of 6 points on AgNW-based transparent heater applying voltage, (**b**–**d**) heating characteristics of transparent heater depending on contact pad design, (**b**) maximum temperature, (**c**) spot average temperature of 6 points, and (**d**) relative standard deviation as heating uniformity.

We analyzed the heating characteristics using an IR camera, as depicted in Figure 3a. In Figure 3a, we measured the heat characteristics of the transparent heater at six points, which represented the two highest temperatures, two middle temperatures, and two lowest temperature spots. To optimize the measurement of the heating characteristics of transparent heaters, we analyzed heating properties by applying 3 V, 5 V, and 7 V. When we applied 7 V to the transparent heater, the AgNWs broke at 170 °C or higher, resulting in no heat generation. Additionally, when applying 3 V to the transparent heater, the heater with line and spot type exhibited very low heating efficiency, as shown in Figure S2. As a result, comparing the heating characteristics of the transparent heater with each shape of the contact pad was challenging due to the high standard deviation shown in Figure S2. To avoid breaking the AgNWs network and to maintain consistent heating characteristics, we selected 5 V as the optimal voltage for comparison and analysis depending on different contact pad shapes. As shown in Figure 3b, an AgNW-based transparent heater with a line-type contact pad reached a maximum temperature of 57 °C. Meanwhile, with the spot-type contact pad, the temperature did not increase as much as the line-type contact pad around 43 °C. However, when applying 5 V to the transparent heater with twist- and parallel-type contact pad electrodes, the maximum temperature increases to 104 °C and 102 °C, respectively. Regarding the temperature response time, which is defined as the time to reach the maximum temperature, we observed the time response time by calculating the slope of the heating characteristics, as shown in Figure S2. For the line contact pad, the temperature response time was 0.138 °C/s, while the parallel contact pad configuration exhibited the highest response time of 0.28 °C/s. In contrast, the spot contact pad had the lowest response time, calculated at 0.08 °C/s. In Figure 3c, we also calculated the average temperature with six spot points. For transparent heaters with line- and spot-type contact pads, the average temperatures were 39 °C and 38 °C, respectively. The average temperature of the transparent heater with twist- and parallel-type contact pads was around 63 °C. These results indicate that both the maximum and average temperatures are influenced by the shape of the contact pad, which facilitates efficient current distribution. Temperature uniformity, an important factor for the stability of transparent heaters, was also analyzed. In Figure 3d, to investigate uniform heating characteristics, we calculate the relative standard deviation (RSD) of heating characteristics using Equation, where the RSD is calculated by dividing the standard deviation by the arithmetic mean [38,39]. The lower RSD value indicates more heat uniformity. For a transparent heater with line- and spot-type contact pads, RSD values were 0.08 and 0.13, respectively. But, for transparent heaters with T- and P-type contact pads, RSD values slightly increased to 0.24 and 0.22, respectively. However, considering the maximum and average temperature, the transparent heaters with T- and P-type contact pad electrodes were suitable. We also measured the transmittance of AgNW-based transparent heaters with various shapes of contact pad electrodes. As shown in Figure S3, AgNW-based transparent heaters with line, spot, and parallel contact pad electrodes have similar specular transmittance, around 85% at 550 nm. However, transparent heaters with T-type contact pads had a lower specular transmittance, about 70%, due to the contact pad electrodes covering the heating surface. Considering the heating characteristics, uniformity, and transmittance, the AgNW-based heater with a P-type contact pad was better compared with other shapes of contact pads.

### 3.2. Heating Characteristics of Transparent Heater by Adjusting Size of Parallel Shape

**Figure 4:**
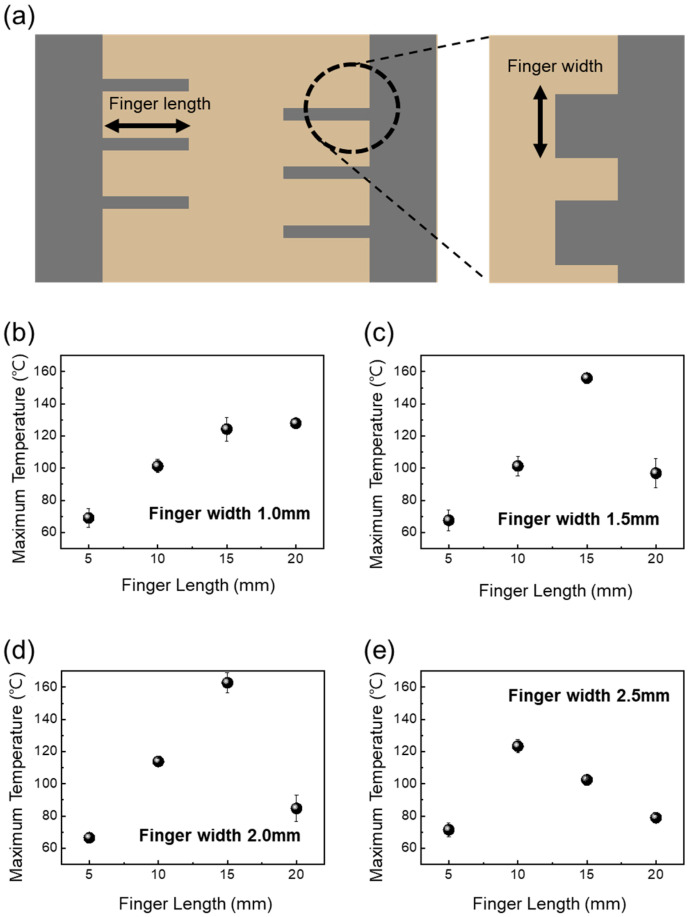
(**a**) Schematic of design of parallel-type contact pad by adjusting finger width and length and maximum temperature of AgNW-based transparent heater depending on finger length with (**b**) finger width 1.0 mm, (**c**) finger width 1.5 mm, (**d**) finger width 2.0 mm, and (**e**) finger width 2.5 mm.

We explored the effect of a parallel shape on the efficient heating characteristics of a transparent heater. To investigate the heating characteristics of transparent heaters with various P-type contact pads, we controlled the length and width of fingers, as illustrated in Figure 4a.

We analyzed how these finger sizes affected the heating characteristics by applying 5 V to the transparent heaters. As shown in Figure 4b–e, we measured the maximum temperature of the transparent heater depending on finger length. Figure 4b shows that for a finger width of 1.0 mm, the maximum temperature increased from 70 °C to 130 °C as the finger length increased. Figure 4c indicates that for a finger width of 1.5 mm, the maximum temperature reached up to 160 °C. However, when the finger width was 1.5 mm and the length was 20 mm, the maximum temperature dropped to 100 °C. As shown in Figure 4d, for a finger width of 2.0 mm, the maximum temperature increased to 165 °C, but with a finger length of 20 mm, the maximum temperature decreased to 80 °C. Also, the maximum temperature was 70 °C at a finger length of 5 mm, but the maximum temperature decreased from 125 °C to 80 °C as the finger length increased from 10 mm to 20 mm. When the finger width of the contact pad was 1.0 mm, the heating temperature varied significantly. The transparent heater with various finger lengths can be categorized into two types, unoverlap and overlap, regardless of finger width, as shown in Figure S4. When the finger width of the contact pad was set to 1.0 mm, the heating characteristics of the transparent heater were analyzed through an IR photograph, as shown in Figure S4. There was significant heating between the fingers of the contact pad as the distance between the two contact pads decreased. However, in the overlap form, the temperature of the transparent heater did not increase as much in the case of the unoverlap form. For the unoverlap form, like the transparent heater with a finger length of 5 mm, the space to the opposite finger of the contact pad was too wide, so it was not efficient to increase the heat on the transparent conducting network. But, as the distance between the fingers of the contact pad became appropriate to a finger length of 15 mm, the heating increased due to smoother electron flow. However, when the distance of the contact pad is too narrow, such as a transparent heater with a finger length of 20 mm, there is not insufficient space for effective heating, despite the rapid flow of electrons. As shown in Figure S4, for a transparent heater with a finger length of 20 mm, the heating characteristics decreased as the finger width increased, indicating that a narrower distance between the contact pads does not allow enough space for effective heating.

**Figure 5:**
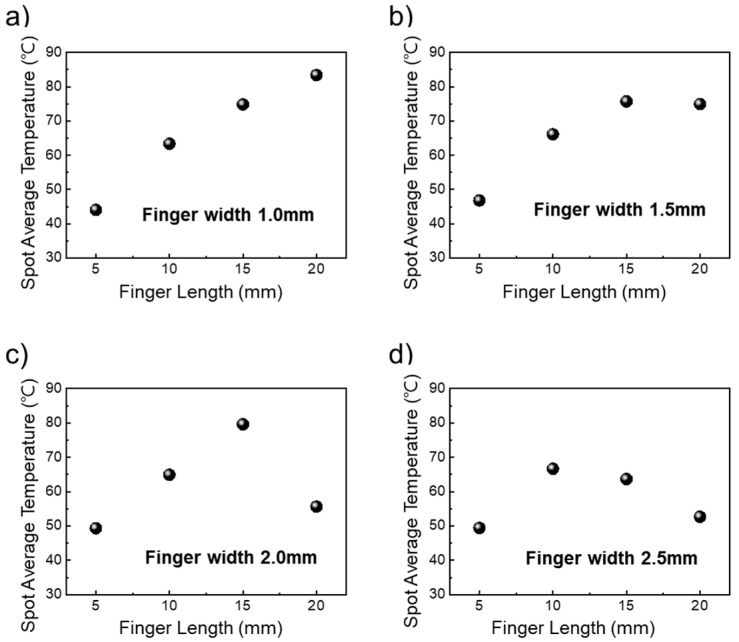
Spot average temperature of 6 points on AgNW-based transparent heater depending on finger length with (**a**) finger width 1.0, (**b**) finger width 1.5 mm, (**c**) finger width 2.0 mm, and (**d**) finger width 2.5 mm.

To compare the spot average temperature of the transparent heater with various contact pads, we measure six points using an IR spot meter. As shown in Figure 5a, for a transparent heater with a finger width of 1.0 mm, the spot average temperature linearly increased from 45 °C to 82 °C as the finger length increased. For a transparent heater with a finger width of 1.5 mm, the spot average temperature also increases from 47 °C to 72 °C, as shown in Figure 4a. However, for finger widths of 2.0 mm and 2.5 mm, when finger length was over 20 mm, the spot average temperatures were similar to those for 5 mm length, approximately 55 °C and 52 °C, respectively.

**Figure 6:**
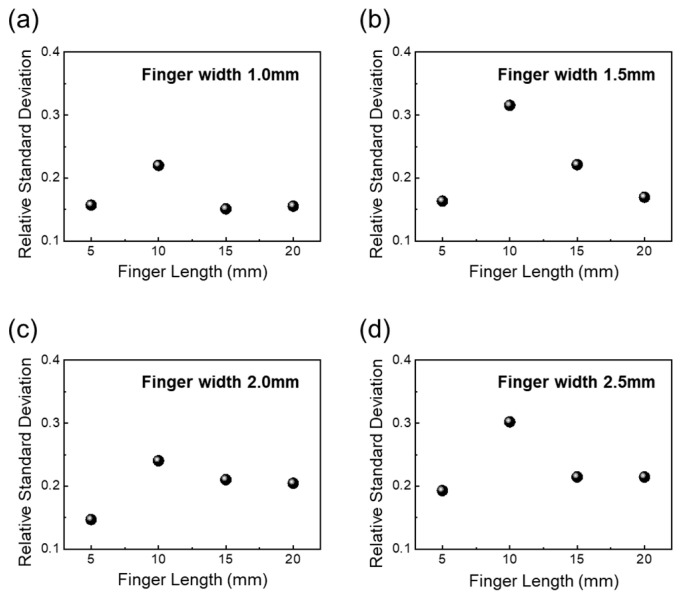
Relative standard deviation of heating characteristics depending on finger length with (**a**) finger width 1.0, (**b**) finger width 1.5 mm, (**c**) finger width 2.0 mm, and (**d**) finger width 2.5 mm.

To compare the heating uniformity of the transparent heater with various contact pads, we calculated the RSD value using spot point temperature. As shown in Figure 6, for a finger width of 1.0 mm, the RSD value is 0.16, 0.22, 0.15, 0.15, with increasing finger lengths from 5 mm to 20 m. For a finger width of 1.5 mm, the RSD value is 0.16, 0.31, 0.22, and 0.17, respectively. The RSD value of a transparent heater with 2.0 mm is 0.14, 0.24, 0.21, 0.24. In the case of a transparent heater with a finger width of 2.5 mm, the RSD value is 0.19, 0.30, 0.21, 0.21. Even with different finger widths, the highest RSD value was observed when the finger length was 10 mm, while nearly the same RSD values were noted for finger lengths of 5 mm, 15 mm, and 20 mm. As shown in Figure S4, we checked the heating mapping by IR photo. Significant joule heating occurred along the main electron flow path on the AgNW network, while relatively less joule heating was generated outside of space between the ends of fingers. Therefore, it was noted that an appropriate distance between the fingers is crucial for increasing the heating temperature and the uniformity of the heating.

### 3.3. Optical Properties of Transparent Heaters Depending on Size of Parallel Shape

**Figure 7:**
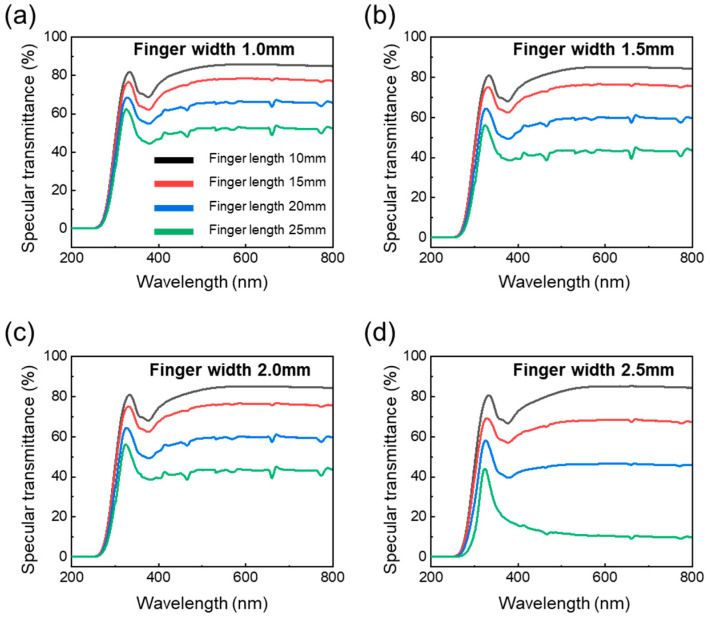
Specular transmittance of AgNW-based transparent heater with parallel type depending on finger length with (**a**) finger width 1.0, (**b**) finger width 1.5 mm, (**c**) finger width 2.0 mm, and (**d**) finger width 2.5 mm.

To investigate the optical properties of AgNW-based transparent heaters, we measured the transmittance of AgNW-based transparent heaters with various P-type contact pads through UV-VIS spectroscopy (UV-2600, Shimadzu, Kyoto, Japan). Figure 7 shows the specular transmittance of an AgNW-based transparent heater with various finger sizes depending on finger width and length. As shown in Figure 6a, for a transparent heater with a finger width of 1.0 mm, the transmittance at 550 nm decreased from 85.6% to 52.4% as the finger length increased because the finger of the contact pad covered more of the surface of the heating area, as depicted by IR photo in Figure S4. As the finger length increased, more of the heater surface, which is crucial for light transmission, became obstructed by metal, reducing overall transmittance. As similar results, for a finger width of 1.5 mm, the transmittance decreased from 84.8% to 45.3% as the finger length increased. For a finger width of 2.0 mm, the transmittance decreased from 85.3% to 43.3%. For a finger width of 2.5 mm, transmittance dramatically decreased from 84.6% to 10.9% because a large space was covered. As finger length increased, the transmittance of the transparent heater decreased because the metal of the finger blocks more of the transparent heating area.

Considering factors for transparent heaters, such as heating temperature, uniformity, and transmittance, it was confirmed that the P-type transparent heater with a finger length of 15 mm and a width of 1.0 mm represented the best contact pad electrode shape and the most optimal overall shape.

## 4. Conclusions

We investigated and optimized various contact pad electrode shapes for AgNW-based transparent heaters. We confirmed that when controlling the shape of the contact pad, the heating characteristics increased even when applying the same power, and we calculated the deviation of the heating characteristics to explore the heating uniformity. The parallel-type contact pad electrode, which shows the most balanced characteristics, achieved the highest maximum temperature of 102 °C, with an RSD value of 0.22, indicating heating uniformity, as well as a high transmittance of 85%. Then, by varying the finger width and length of the parallel-type contact pad, the optimal contact pad electrode for the transparent heater was a parallel-type contact pad with a finger length of 10 mm and a width of 1.0 mm, which had a maximum temperature of 120 °C, an RSD value of 0.15, and over 80% of transmittance.

We expect that the implementation of this optimized contact pad shape will contribute to the advancement of applications such as antifog glass, smart windows, and agricultural technology. This study contributes to the fundamental advancement of transparent heater technology through simple contact pad design without engineering transparent conducting materials, providing appropriate solutions to problems related to heating efficiency, uniformity, and optical properties.

## Data Availability

The data presented in this study are available on request from the corresponding author. The data are not publicly available due to privacy.

## Supplementary Materials

The following supporting information can be downloaded at https://www.mdpi.com/article/10.3390/nano14211735/s1, Figure S1. Electrical and thermal measurement for transparent heater (a) set up for thermal characteristics; (b) photograph of electrical measurement by portable 4-point probe; Figure S2: Heating characteristics of transparent heater with various contact pad electrodes depending on applied voltage (3 V, 5 V, 7 V) and IR photo of (a) line type, (b) spot type, (c) twist type, (d) parallel type; Figure S3: Specular transmittance of transparent heater with various contact pad electrodes; line, spot, twist, parallel type; Figure S4: Heating characteristics of transparent heater with parallel type by adjusting finger length (a–d) finger width 1.0 mm, (e–h) finger width 1.5 mm, (i–m) finger width 2.0 mm, (n–q) finger width 2.5 mm.
