# Supplementary material for: Optimization of Contact Pad Design for Silver Nanowire-Based Transparent Heater to Improve Heating Characteristics
Source: Nanomaterials (Basel). 2024 Oct 29;14(21):1735. doi: 10.3390/nano14211735 (PMC11547844; doi:10.3390/nano14211735)
Supplement: Supplementary file 1 [file nanomaterials-14-01735-s001.zip › nanomaterials-3268657-supplementary.pdf]

Optimization of Contact Pad Design for Silver Nanowire-based Transparent Heater to Improve Heating Characteristics

Seo Bum Chu [1,†], Yoohan Ma [1,2,†], Jinwook Jung [1,2], Sungjin Jo [3], Dong Choon Hyun [4], Jae-Seung Roh [1,5], Jongbok Kim [1,2,5,*], Dongwook Ko [5,*]

[1] Department of Materials Science and Engineering, Kumoh National Institute of Technology, Gumi, Gyeongbuk 39177, Republic of Korea; csb5453@naver.com (S.B.C.), john931023@gmail.com (Y.M.), jkim3161@gmail.com (J.J.) jsroh@kumoh.ac.kr (J.-S.R), jbkim@kumoh.ac.kr (J.K.), duko1293@gmail.com (D.K.)

[2] Department of Energy Engineering Convergence, Kumoh National Institute of Technology, Gumi, Gyeongbuk 39177, Republic of Korea; john931023@gmail.com (Y.M.), jkim3161@gmail.com (J.J.), jbkim@kumoh.ac.kr (J.K.)

[3] School of Architectural, Civil, Environmental, and Energy Engineering, Kyungpook National University, Daegu 41566, Republic of Korea; sungjin@knu.ac.kr (S.J.)

[4] Department of Polymer Science and Engineering, Kyungpook National University, Daegu 41566, Republic of Korea; dong.hyun@knu.ac.kr (D.C.H.)

[5] Advanced Materials Research Center, Kumoh National Institute of Technology, Gumi, Gyeongbuk 39177, Korea; jsroh@kumoh.ac.kr (J.-S.R), jbkim@kumoh.ac.kr (J.K.), duko1293@gmail.com (D.K.)

*** Correspondence: jbkim@kumoh.ac.kr (J.K.); duko1293@gmail.com (D.K.); Tel: +82-54-478-7748 (J.K.)**

† These authors contributed equally to this work.

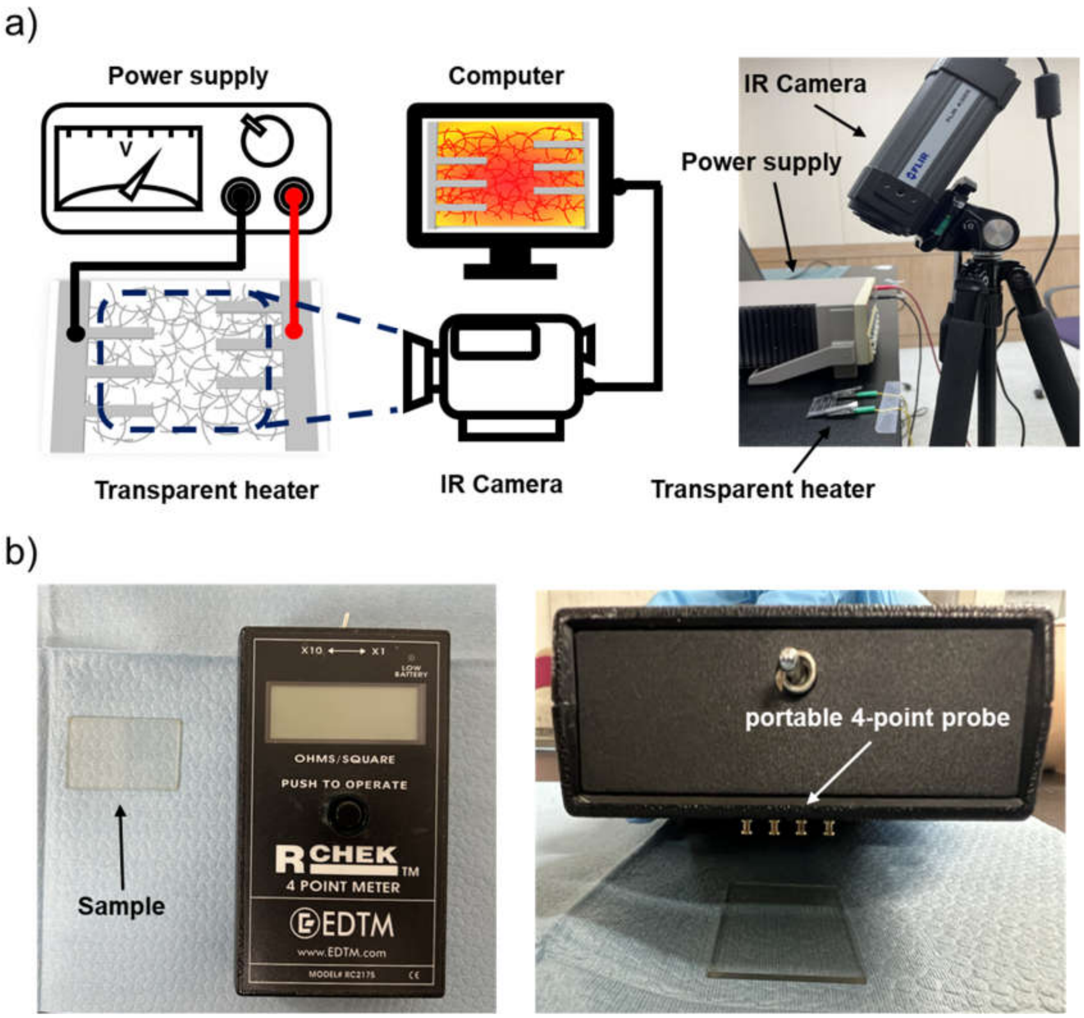

Figure S1. electrical and thermal measurement for transparent heater a) set up for thermal characteristics b) photograph of electrical measurement by portable 4-point probe

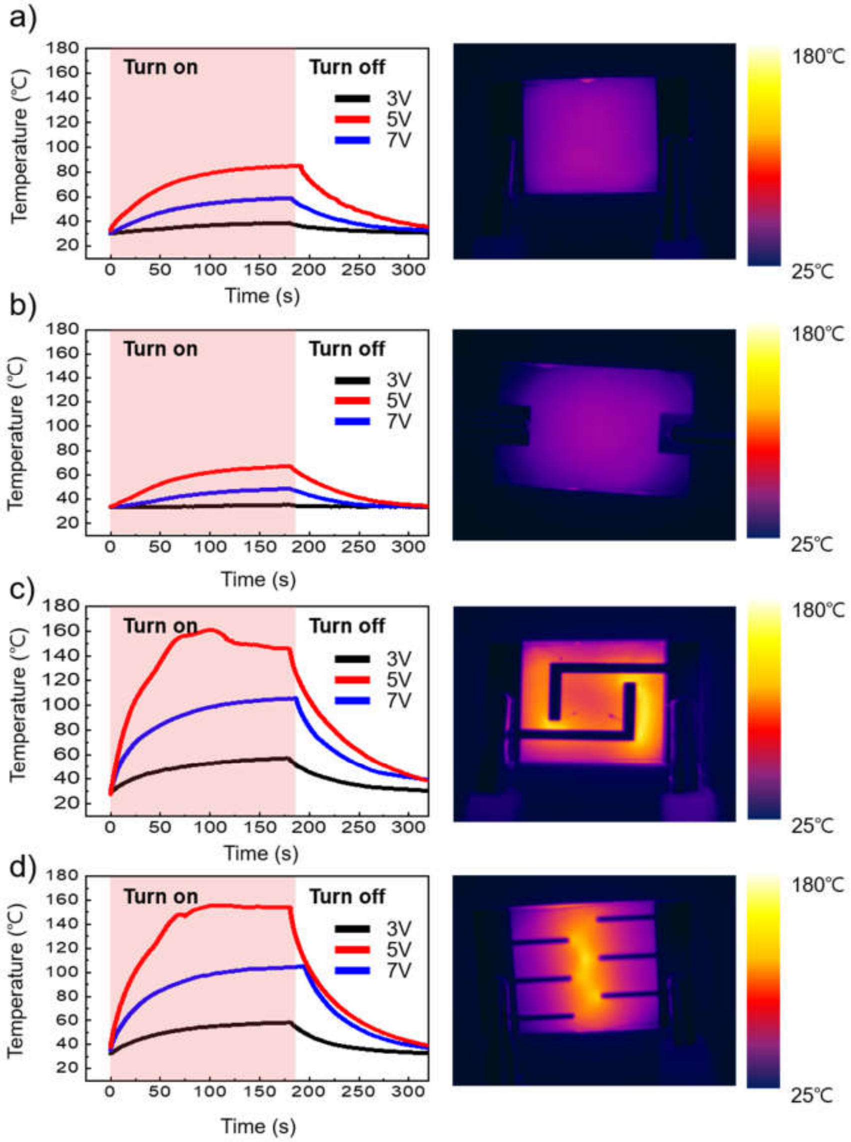

Figure S2. Heating characteristics of transparent heater with various contact pad electrodes depending on applied voltage (3 V, 5V, 7V) and IR photo of a) line type, b) spot type, c) twist type, d) parallel type

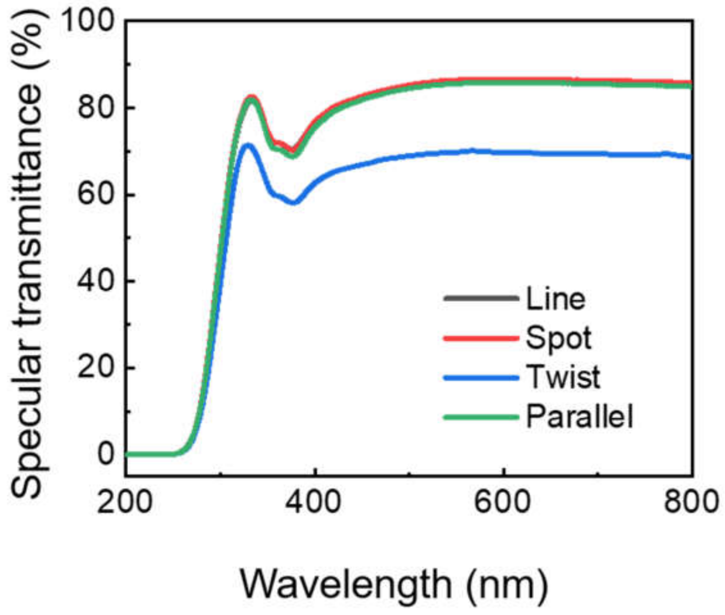

Figure S3. Specular transmittance of transparent heater with various contact pad electrodes; line, spot, twist, parallel type

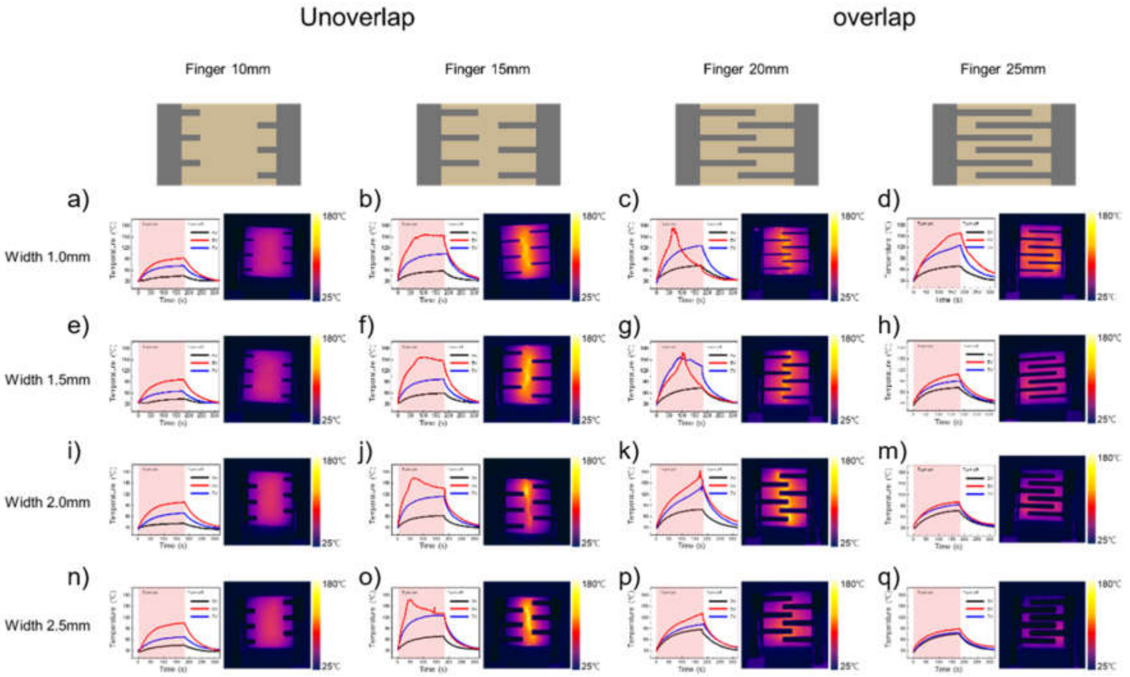

Figure S4. Heating characteristics of transparent heater with parallel type by adjusting finger length a)-d) finger width 1.0 mm, e)-h) finger width 1.5 mm, i)-m) finger width 2.0mm, n)-q) finger width 2.5 mm
